# Perlecan Domain V Induces VEGf Secretion in Brain Endothelial Cells through Integrin α_5_β_1_ and ERK-Dependent Signaling Pathways

**DOI:** 10.1371/journal.pone.0045257

**Published:** 2012-09-17

**Authors:** Douglas N. Clarke, Abraham Al Ahmad, Boyeon Lee, Christi Parham, Lisa Auckland, Andrezj Fertala, Michael Kahle, Courtney S. Shaw, Jill Roberts, Gregory J. Bix

**Affiliations:** 1 Department of Molecular & Cellular Medicine, Texas A&M Health Science Center, College Station, Texas, United States of America; 2 Center for Vascular Biology Research, Department of Surgery, Beth Israel Deaconess Medical Center, Harvard Medical School, Boston, Massachusetts, United States of America; 3 Department of Dermatology and Cutaneous Biology, Thomas Jefferson University, Philadelphia, Pennsylvania, United States of America; 4 Department of Neuroscience and Experimental Therapeutics, Texas A&M Health Science Center, College Station, Texas, United States of America; 5 Sanders-Brown Center on Aging, Department of Anatomy and Neurobiology, and Department of Neurology, University of Kentucky, Lexington, Kentucky, United States of America; Indiana University School of Medicine, United States of America

## Abstract

Perlecan Domain V (DV) promotes brain angiogenesis by inducing VEGF release from brain endothelial cells (BECs) following stroke. In this study, we define the specific mechanism of DV interaction with the α_5_β_1_ integrin, identify the downstream signal transduction pathway, and further investigate the functional significance of resultant VEGF release. Interestingly, we found that the LG3 portion of DV, which has been suggested to possess most of DV’s angio-modulatory activity outside of the brain, binds poorly to α_5_β_1_ and induces less BEC proliferation compared to full length DV. Additionally, we implicate DV’s DGR sequence as an important element for the interaction of DV with α_5_β_1_. Furthermore, we investigated the importance of AKT and ERK signaling in DV-induced VEGF expression and secretion. We show that DV increases the phosphorylation of ERK, which leads to subsequent activation and stabilization of eIF4E and HIF-1α. Inhibition of ERK activity by U0126 suppressed DV-induced expression and secretion of VEGR in BECs. While DV was capable of phosphorylating AKT we show that AKT phosphorylation does not play a role in DV’s induction of VEGF expression or secretion using two separate inhibitors, LY294002 and Akt IV. Lastly, we demonstrate that VEGF activity is critical for DV increases in BEC proliferation, as well as angiogenesis in a BEC-neuronal co-culture system. Collectively, our findings expand our understanding of DV’s mechanism of action on BECs, and further support its potential as a novel stroke therapy.

## Introduction

Stroke is the leading cause of long term disability and a major cause of death within the United States, with an average fatality rate slightly over 134,000 deaths/year and an overall cost of over $7 billion/year [1]. A better understanding of the mechanisms underlying brain self-repair after stroke constitutes an essential research priority [2] and could lead to improving brain reparative processes. Following cerebral ischemia, there is rapid proteolysis of the extracellular matrix (ECM) as well as dramatic changes in the expression of ECM receptors, cell-bound integrins, in the infarct core and ischemic penumbra regions [3]–[5]. Within this context, we hypothesized that the brain ECM may play a role in post-stroke brain repair.

Several ECM components have C-terminal fragments that possess biological activity following proteolytic cleavage from their parent protein [6], [7]. Perlecan, an ECM heparan sulfate proteoglycan, contains 5 distinct protein domains (Domains I-V), each containing protein subunits with structural homology to other proteins [8]. Domain V (DV), the C-terminal fragment of perlecan, has anti-angiogenic activity outside of the brain following cleavage from perlecan, and therefore is also referred to as endorepellin [9], [10]. DV is an 82 kDa peptide composed of three laminin-like globular (LG1, 2, and 3) subunits, each separated by two epidermal growth factor (EGF, termed EGF1–4 from N terminus to C terminus) subunits. Importantly, LG3, the 24 kDa C-terminal portion of DV, has been reported to be responsible for DV’s anti-angiogenic activity [11].

Until recently, the only DV/LG3 receptor described in endothelial cells was the collagen receptor α_2_β_1_ integrin [12]. Interestingly, although equal or significantly lower nanomolar concentrations of LG3 (compared to DV) are required for α_2_β_1_ integrin-mediated suppression of angiogenesis, LG3 binds to the α_2_β_1_ integrin (specifically, the α_2_ ligand binding domain) with significantly lower affinity (K*_d_* of 1 µM) than does full length DV (K*_d_* of 80 nM), suggesting a much more complex relationship between DV, its LG3 component, the α_2_β_1_ integrin, and inhibition of angiogenesis [11]. Indeed, a more complex relationship has been suggested whereby the LG1 and LG2 components of intact DV bind to VEGFR1 or VEGFR2 and the LG3 portion simultaneously binds to α_2_β_1_ resulting in transcriptional repression of VEGF [13].

It has been shown that DV and LG3 are actively and persistently cleaved from full length perlecan after stroke [14], [15] by a number of proteases including BMP-1/Tolloid-like metalloproteases and cathepsin-L [16], [17]. We recently demonstrated that DV is unexpectedly pro-angiogenic both *in vitro* and *in vivo* after experimental focal cerebral ischemia [14]. This pro-angiogenic effect occurs in brain microvessels, where the α_2_β_1_ integrin is largely absent [18], [19], and is instead driven by VEGF released following direct interaction of DV with the fibronectin receptor α_5_β_1_ integrin. However, the mechanisms by which DV interacts with α_5_β_1_ and induces VEGF expression, as well as the potential of LG3 to bind α_5_β_1_ and/or exert a pro-angiogenic effect in brain endothelial cells (BECs), remain unclear. Therefore, the present study aimed to: 1) Further define the interaction of DV with the α_5_β_1_ integrin, 2) Evaluate LG3 binding to α_5_β_1_ integrin and determine whether it also exerts pro-angiogenic activity on BECs, 3) Identify the signaling pathways activated downstream of DV’s interaction with the α_5_β_1_ integrin that results in VEGF release, and 4) Further demonstrate the functional significance of DV’s induction of VEGF on BEC cell physiology. Collectively, our findings expand our understanding of DV’s mechanism of action on BECs, and further support its potential as a novel stroke therapy.

## Results

### DV Binding to α_5_β_1_ Integrin is Partially Mediated by its DGR Sequence

**Figure 1:**
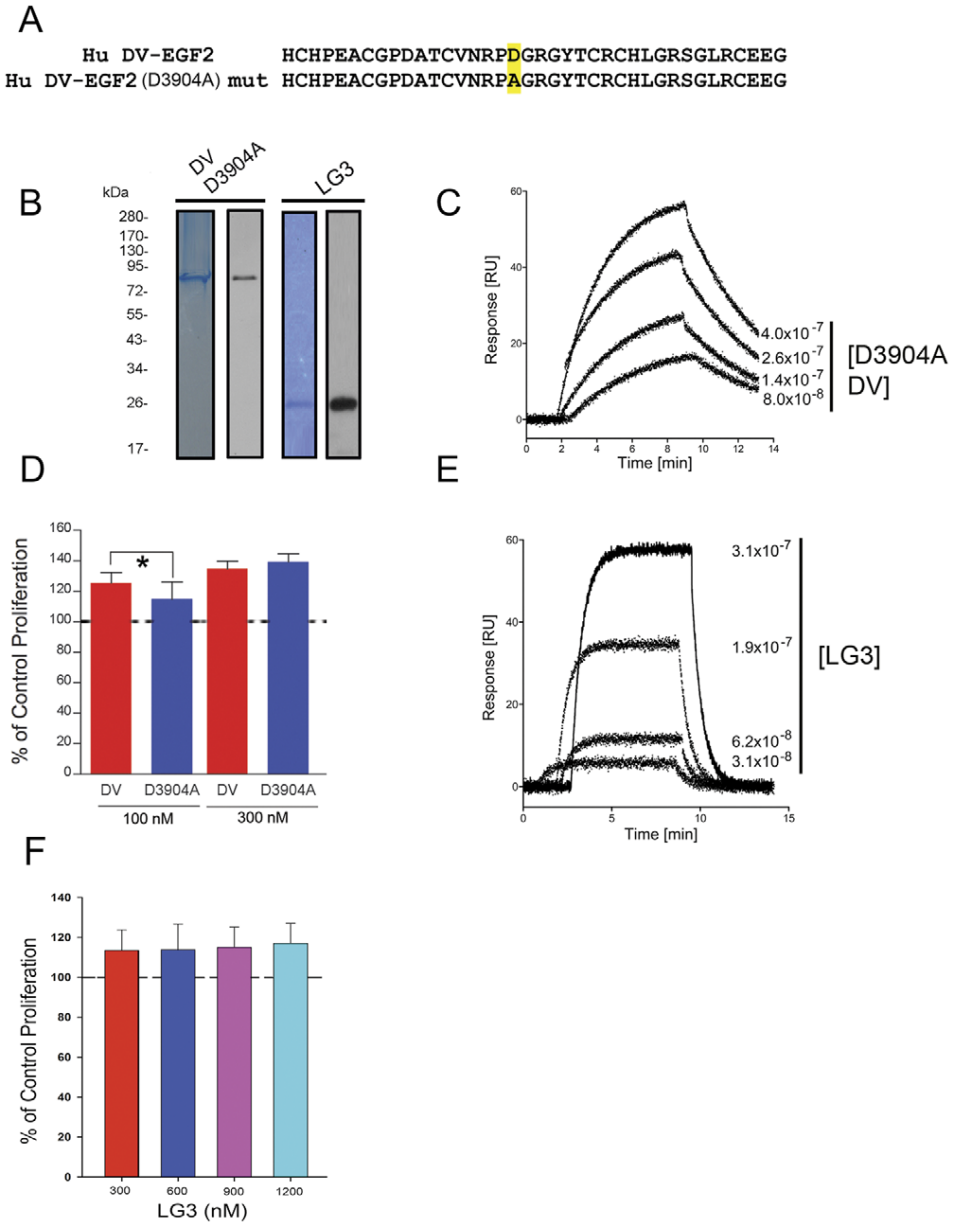
DV binding to α_5_β_1_ is partially mediated through its DGR sequence. (A) Sequence schematic of the second EGF repeat within DV demonstrating the exact location, highlighted in yellow, where D3904A DV was mutated. (B) Coomassie stain of SDS PAGE of D3904A DV and LG3 protein preparations (left image for each as labeled) and anti-his immunoblot (right image for each as labeled) to recognize the 6xHis-tag present in the recombinant purified D3904A DV and LG3 (DV C-terminal fragment). In both analyses, D3904A DV yields a single 82 kDa band as does wild-type DV (not shown), and LG3 yields a single 25 kDa band. (C) Optical biosensor traces showing the association and dissociation of D3904A DV with immobilized α_5_β_1_ integrin at the concentrations listed (RU = relative units). (D) Quantification of proliferation of BECs after 24 h ± wild-type DV or D3904A DV at 100 nM or 300 nM concentrations in serum starved media as measured via MTS assay. N = 3, *P<0.05 at 100 nM, *P* = 0.06 at 300 nM. (E) Optical biosensor traces showing the association and dissociation of LG3 with immobilized α_5_β_1_ integrin at the concentrations listed (RU = relative units). (F) LG3 (different concentrations of LG3 as labeled) BEC 24 h proliferation assay.

DV binds to the α_5_β_1_ integrin with a K*_d_* of 160 nM [14]. One possibility for how this interaction might occur is that human DV contains a single DGR amino acid sequence (amino acid number 3904–3906) within its second EGF-like repeat (EGF2) immediately before LG2 [20], [21]. DGR sequences are known to bind integrins and spontaneous, non-enzymatic isomerization by dehydration of the aspartic acid (D) to iso-D, results in a novel iso-DGR integrin binding motif for α_5_β_1_ and α_v_β_3_ integrins [22], [23]. In order to determine the importance of DV’s DGR sequence for DV’s interaction with α_5_β_1_ integrin, as well as its importance for DV’s proangiogenic activity, we generated a DV mutant that has an ‘A’ in the place of the ‘D’ in the DGR sequence (D3904A DV, Figure 1A). D3904A DV did not deleteriously effect protein purification (Figure 1B). Optical biosensor analysis demonstrated that D3904A DV bound to α_5_β_1_ integrin with significantly lower affinity than wild-type DV, as we measured a K*_d_* of 3.7×10^−7^±3.5×10^−9^ M, K*_on_* of 6.0×10^5^±4.5×10^4^/M-s and K*_off_* of 2.2×10^−1^±1.9×10^−2/^s (as compared to K*_d_* of 1.6×10^−7^±7.2×10^−8 ^M, K*_on_* of 3.8×10^6^±2.7×10^5^/M-s, and K*_off_* of 7.2×10^−1^±1.1×10^−1^/s for wild-type DV [14]) (Figure 1C). Because we observed a significant difference in our K*_d_* values of WT DV and D3904A DV, we next asked whether this mutation had any functional significance in our proliferation assays.

The decrease in affinity was accompanied by lower BEC proliferative activity of D3904A DV compared to wild-type DV (Figure 1D). We should note; however, that although we observed this lower proliferative affect at 100 nM (*P<0.05), no difference was observed at higher concentrations between wild-type DV and D3904A DV, suggesting we had reached saturating concentrations of WT DV and D3904A DV.

Next, as previous work had demonstrated that LG3 had significantly less affinity for the α_2_ integrin compared to WT DV [11], we were interested in determining whether this was also the case for LG3’s affinity for α_5_β_1_ integrin, and what effect, if any, it had of BEC proliferation compared to WT DV. Biosensor analysis demonstrated that LG3 bound with a much lower affinity (compared to WT DV) to α_5_β_1_ integrin with a measured K*_d_* of only 1.0×10^−3^±7.0×10^−5^ M, a K*_on_* of 1.6×10^3^±5.8×10^1^/M-s, and a K*_off_* of 1.7±4.3×10^−2^/s (Figure 1E). Furthermore, LG3 was less proliferative in BEC compared to wild-type DV (Figure 1F). Indeed, LG3 concentrations as high as 1200 nM were still less effective than 300 nM DV in stimulating BEC proliferation. Additionally, unlike WT DV, LG3 did not elicit a proliferative dose response in BECs.

### DV Induces Akt Phosphorylation through a PI3K-dependent Mechanism

To investigate the intracellular signaling cascade involved with DV-induced VEGF release [14], we investigated the temporal phosphorylation of several cell signal transduction components known to be involved in VEGF regulation, such as Akt and ERK [24], and whether inhibition of these components could inhibit DV-induced VEGF release.

**Figure 2:**
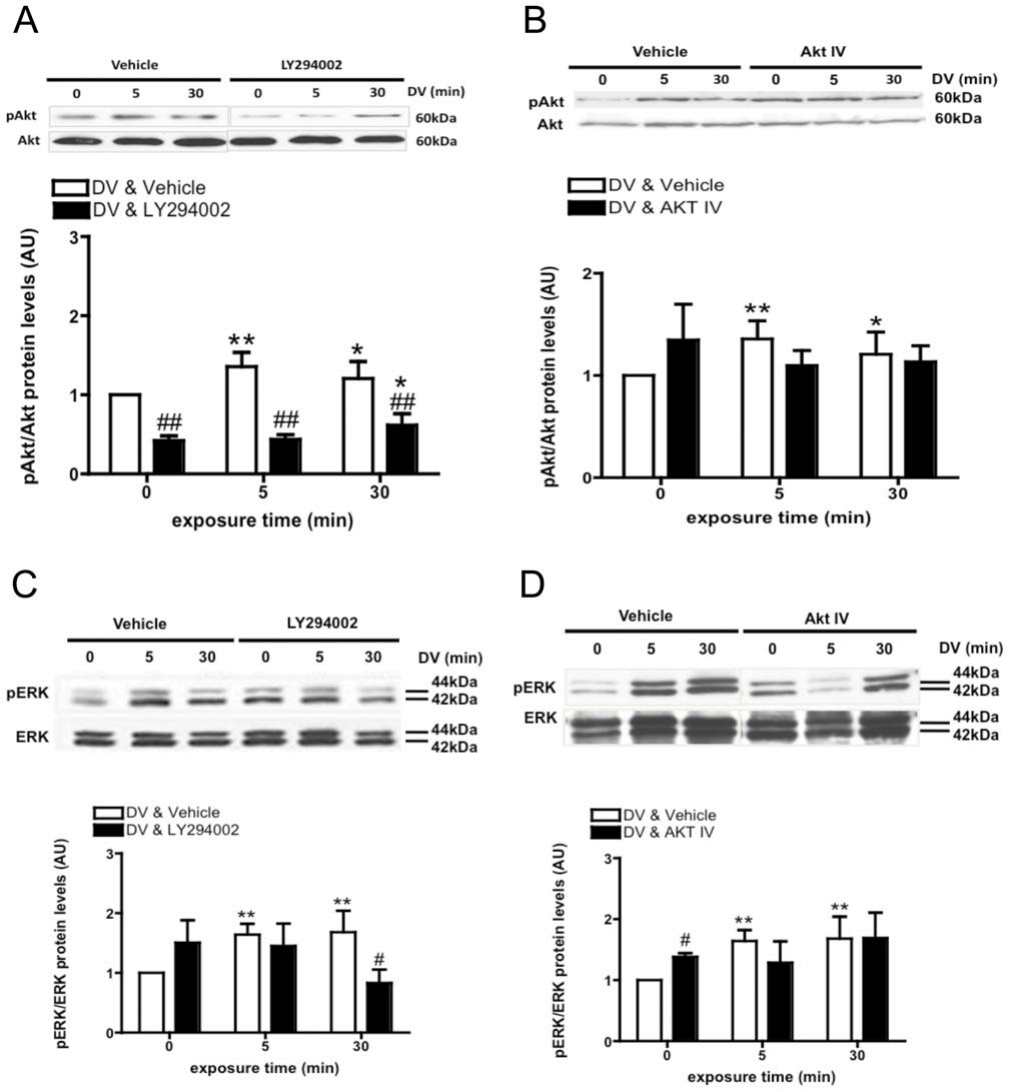
DV increases both Akt and ERK phosphorylation. (A) Immunoblots (upper) and OD quantification (lower) for phosphorylated Akt (pAkt) and pan-Akt (Akt) from BECs in the presence of DV and/or 10 µM LY294002 (PI3K inhibitor). N = 3, *P<0.05 and **P<0.01 versus 0 min timepoint, ^##^P<0.01 versus DV-only treated group (vehicle) (B) Immunoblots (upper) and OD quantification (lower) for pAkt and Akt in BECs treated with 10 µM AktIV (Akt inhibitor). N = 3, *P<0.05 and **P<0.01 versus 0 min timepoint. (C) Immunoblots (upper) and OD quantification (lower) for phosphorylated ERK (pERK) and pan-ERK (ERK) in BECs treated with 10 µM LY294002. N = 3, **P<0.01 versus 0 min timepoint, ^#^ *P*<0.01 versus DV-only treated group (vehicle). (D) Immunoblots (upper) and OD quantification (lower) for phosphorylated ERK (pERK) and pan-ERK (ERK) in BECs treated with 10 µM AktIV. N = 3, **P<0.01 versus 0 min timepoint, ^#^ *P*<0.01 versus DV-only treated group (vehicle).

Treatment of BECs with DV resulted in a significant increase in Akt phosphorylation (pAkt) at both 5 and 30 min as measured by western blot (**P<0.01 and *P<0.05 respectively, Figure 2A). (It should be noted, however, that under some circumstances elevated basal pAkt was unavoidable). Because we observed elevated basal pAkt in some scenarios, we performed a cell-based ELISA and confirmed DV induces p-Akt (data not shown). In the presence of 10 µM LY294002, a selective PI3K inhibitor, we observed a significant delay in DV-induced Akt phosphorylation, resulting in a significant increase in pAKT only at 30 min. We further investigated decreased activation in Akt phosphorylation in the presence of 10 µM Akt IV, another inhibitor of Akt activity (Figure 2B). Akt IV blocks the ATP binding site of a kinase upstream to AKT yet downstream to PI3K. In the presence of Akt IV, basal pAkt levels were increased compared to the untreated group. However, addition of DV to BECs in the presence of Akt IV did not cause any change in pAkt at 5 or 30 min.

### Akt Inhibition by Pharmacological Agents Prevents DV-induced ERK Phosphorylation

PI3K/Akt and MEK-ERK signaling pathways have previously been shown to cross-talk [25]. Therefore, we investigated the impact of Akt phosphorylation on ERK phosphorylation, following DV exposure, by investigating changes in phosphorylated ERK levels in the presence of LY294002 or Akt IV (Figure 2C, D). In the absence of these inhibitors, DV rapidly induced ERK phosphorylation compared to basal levels. In the presence of 10 µM LY294002 there was an increase in phosphorylated ERK (pERK), suggesting that basal PI3K/Akt activity may partially inhibit the ERK signaling pathway [24]. In the presence of LY294002, the addition of DV did not induce any further increases in pERK levels at 5 min, yet at 30 min, we observed a significant decrease in pERK. As observed with LY294002, Akt IV increased pERK levels under basal conditions (Figure 2D). Although we observed a slight decrease in pERK levels at 5 min, pERK levels rose above baseline at 30 min.

### DV Induction of VEGF Gene Expression and Secretion is Driven through a ERK1/2 Dependent Pathway

**Figure 3:**
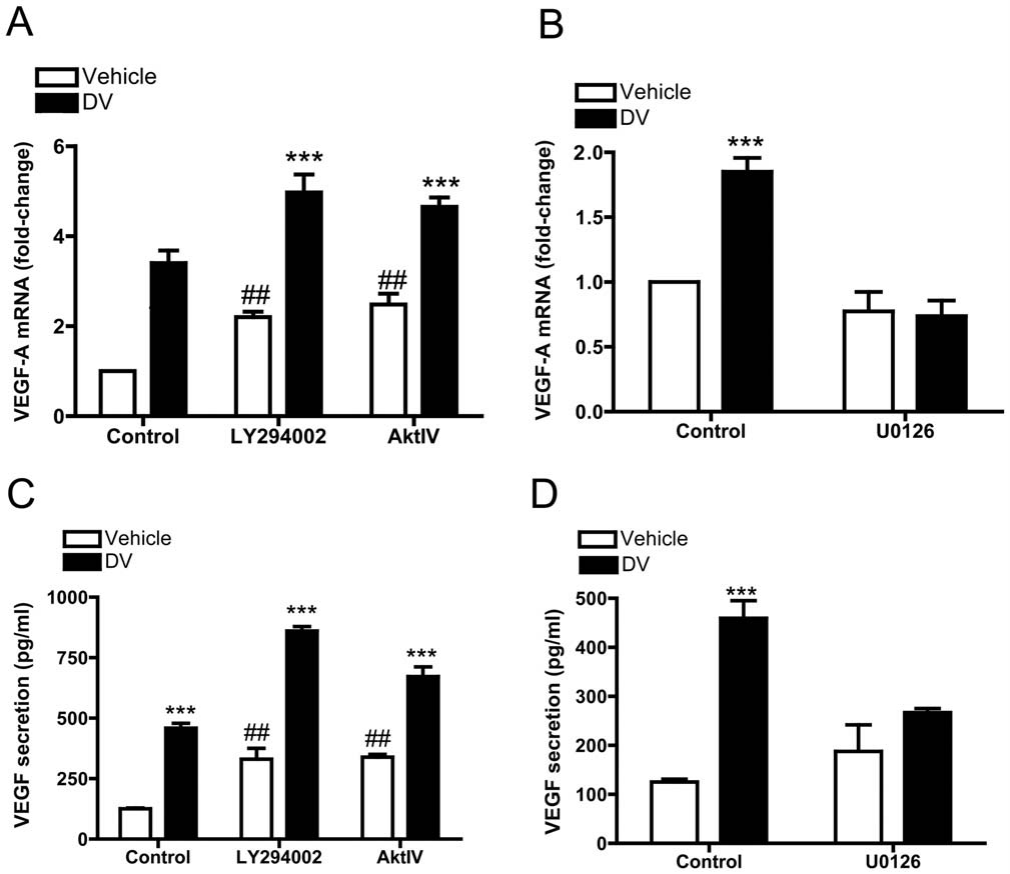
DV increases VEGF-A at mRNA and protein level through an ERK-dependent pathway. (A) qPCR analysis of VEGF-A mRNA in BECs +/− DV and +/−10 µM LY294002 or 10 µM AktIV. GAPDH was used as internal control. N = 3, ***P<0.001 in comparison between DV-treated and untreated cells, ^##^ *P*<0.01 in comparison between inhibitors and untreated groups. (B) Similar analysis as in (A) demonstrating that 10 µM U0126 (MEK inhibitor) treatment significantly blunted DV-induced VEGF-A mRNA up-regulation. N = 3. ***P<0.001 in comparison between DV-treated and untreated cells. (C) Secreted VEGF ELISA profile from BECs following treatment with DV, LY294002, or AktIV alone or in combination Note the significant up-regulation of secreted VEGF following DV, LY294002 or AktIV treatment. N = 3, ***P<0.001 in comparison between DV-treated and untreated cells, ^##^ *P*<0.01 in comparison between LY and AktIV treated groups versus untreated group. (D) U0126 treatment significantly inhibited VEGF secretion from BECs following DV treatment. N = 3. ***P<0.001 in comparison between DV-treated and untreated cells.

BDNF-induced activation of PI3K/AKT and MEK pathways in endothelial cells leads to the induction of VEGF secretion. Therefore, because DV induces VEGF expression and secretion in BECs and also activates Akt and ERK, we next investigated whether DV-inducted VEGF expression and secretion could be mediated by or involve Akt and ERK activation. Quantitative PCR was used to detect VEGF-A expression with and without DV treatment (1.5 h), in the absence or presence of Akt or ERK inhibitors. LY294002 or Akt IV alone increased VEGF-A mRNA basal levels. To our surprise, neither inhibitor was efficient in blocking DV-induced VEGF-A mRNA up-regulation (Figure 3A). Instead, we observed a significant increase in VEGF mRNA expression when cells were treated with both DV and either inhibitor. However, U0126 (10 µM) was able to significantly inhibit DV-induced VEGF-A mRNA expression (Figure 3B). Next, VEGF-A ELISA was performed to detect secreted VEGF-A from BECs supernatants with and without DV treatment, in the absence or presence of Akt or ERK inhibitors. In accordance with our results that Akt inhibitors increased VEGF expression, we also observed an increase in VEGF secretion and an additive increase with the addition of DV (Figure 3C). However, significant inhibition of DV-induced VEGF secretion was obtained with 10 µM U0126 (Figure 3D), further implicating ERK activation in DV induced VEGF secretion.

### DV Induces VEGF Expression through eIF4E- and c-Jun Dependent Pathways

**Figure 4:**
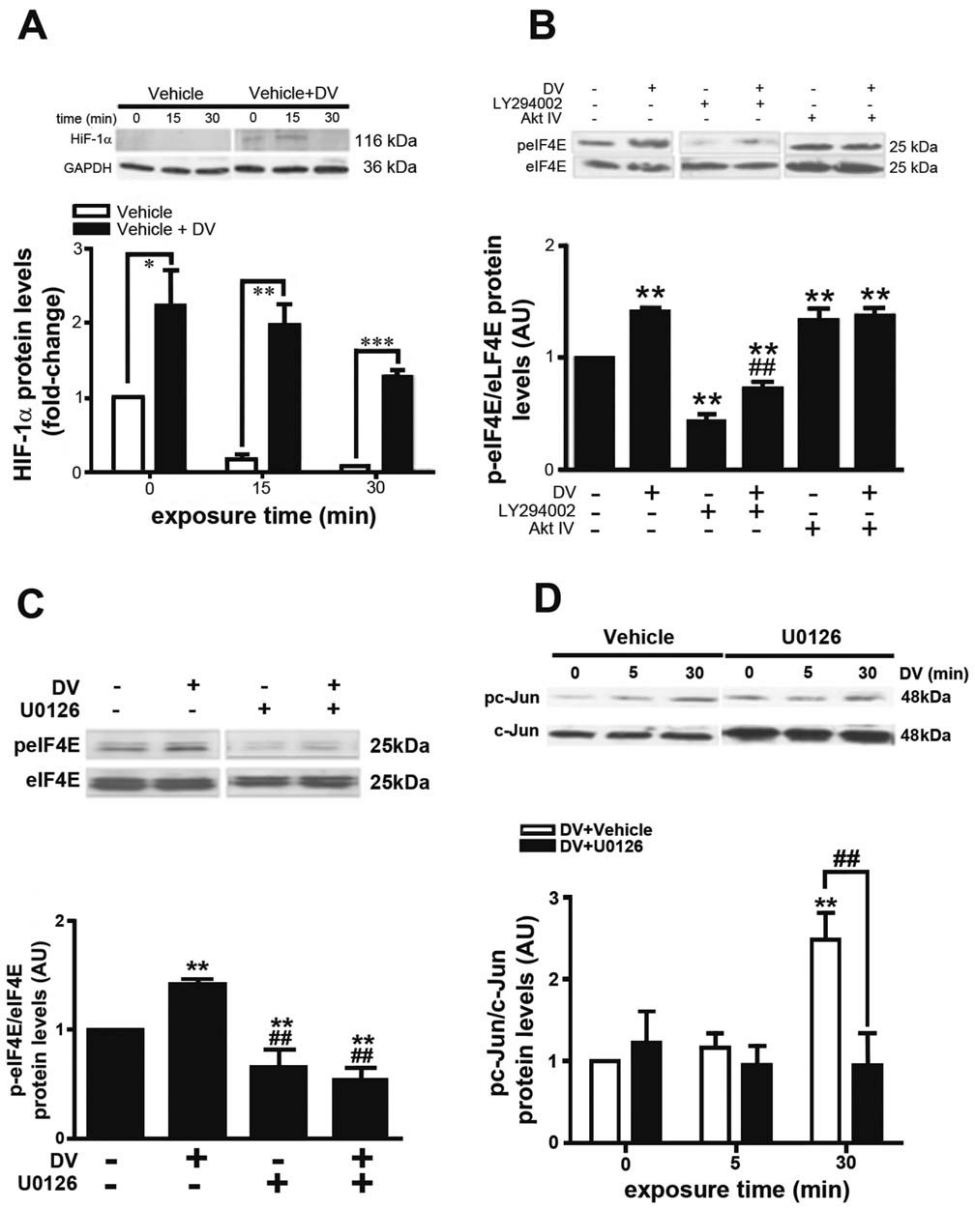
DV induces HIF-1α and increases eukaryotic initiation factor 4E. (A) HIF-1α immunoblot (upper) and OD quantification (lower) following DV exposure. Note the transient HIF-1α band at 5 minutes. N = 3, *P<0.05, **P<0.01 and ***P<0.001 in comparison between vehicle and DV treated cells. (B) Immunoblots (upper) of eukaryotic initiation factor 4E phosphorylation (peIF4E) and total eIF4E after treatment of BECs with combinations of DV, LY294002 and AktIV as labeled. Cells were treated with DV for 5 min. OD quantification (lower) of peIF4E as normalized to total eIF4E. N = 3, **P<0.01 versus untreated cells, ^##^ *P*<0.01 within LY294002 treated-group (C) Immunoblots (upper) of peIF4E and eIF4E from BECs treated with combinations of DV and U0126 as labeled demonstrating that U0126 significantly abolished DV-induced peIF4E hyperphosphorylation as OD quantified (lower). Cells were treated with DV for 5 min. N = 3, **P<0.01 versus control, ^##^ *P*<0.01 versus DV-treated group. (D) Immunoblots (upper) of phosphorylated c-jun (pc-jun) and total c-jun from BECs treated with DV +/− U0126 demonstrating that U0126 significantly inhibited DV-induced c-Jun phosphorylation as OD quantified (lower). Note the significant increase after 30 min following DV treatment. N = 3, **P<0.01 in comparison to untreated cells, ^##^ *P*<0.01 between U0126-treated and untreated cells.

VEGF gene expression is highly convergent on the hypoxia-inducible factor-1 (HIF-1) pathway. While most reports have investigated HIF-1α under hypoxic conditions, there is a growing body of evidence that growth factors can activate HIF-1α under normoxic conditions and cause an increase in VEGF expression that involves ERK. Because our observations suggest that DV is signaling through ERK, we hypothesized that the ability of DV to trigger VEGF expression may also involve activation of HIF-1α in BECs (Figure 4A). Addition of vehicle alone to BECs did not induce HIF-1α stabilization. However, DV treatment rapidly induced HIF-1α stabilization, as we noted its immunopositive band after 5 min of treatment. This stabilization appeared transient as HIF-1α immunoreactivity mostly vanished in cells treated with DV for 30 min.

A possible mechanism by which HIF-1α is stabilized under normoxic conditions may be via its translation through a mammalian target of rapamycin (mTOR)-dependent pathway in which phosphorylation of eukaryotic initiation factor 4E (eIF4E) is a key step. Therefore, we examined changes in eIF4E phosphorylation (peIF4E) in BECs by western blot (Figure 4B). DV treatment alone significantly increased detectable peIF4E (**P<0.01) whereas treatment with LY294002 (10 µM) alone significantly decreased peIF4E levels compared to untreated control BECs. The latter result could be due to a minor contribution of the PI3K/Akt-independent pathway. To confirm this observation, we measured changes in peIF4E levels in the presence of Akt IV inhibitor. Surprisingly, Akt inhibition alone significantly increased peIF4E levels under basal conditions to levels similar to DV treatment alone, suggesting a negative effect of Akt towards the signaling pathway driven by DV. Application of DV with Akt IV did not further increase peIF4E suggesting that DV activity or inhibition of Akt activity may be efficient enough to reach maximum levels of peIF4E levels. Conversely, treatment of BECs with U0126 (Figure 4C) significantly blocked DV-induced peIF4E phosphorylation. Lastly, we investigated changes in c-Jun phosphorylation, a protein belonging to the AP-1 complex that induces VEGF through an ERK-dependent mechanism (Figure 4D). DV treatment significantly increased phospho-c-Jun at 30 min that was significantly blocked by U0126.

### DV-induced VEGF Secretion Induces Functional Changes in Angiogenesis in BECs in vitro

**Figure 5:**
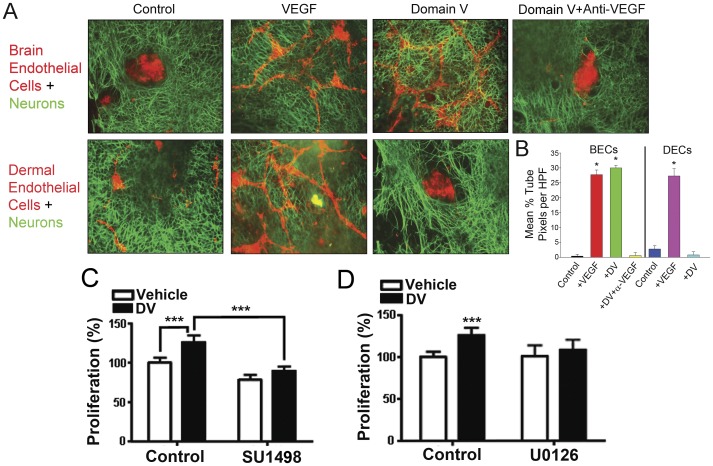
DV enhances brain endothelial cell capillary tube-like structure formation in neuronal co-culture in a VEGF-dependent fashion. (A) BECs or mouse dermal endothelial cells (DECs, red) were cultured on a bed of rat cerebellar granule neurons (green) in serum free medium for 6 hours +/− PBS vehicle control, VEGF, DV, or DV+VEGF neutralizing antibody. Scale bar is 50 µm. (B) Quantification of capillary tube-like structures as in A. *P<0.05 compared to corresponding control. (C) and (D) Quantification of percent BEC proliferation after 24 h (in reference to control, PBS vehicle treated condition arbitrarily set at 100%) treated as labeled. ***P<0.001.

VEGF constitutes an important factor eliciting endothelial cell tube formation, permeability [26], and proliferation [27], [28]. Therefore, we investigated changes in these biological features as a direct readout of DV activity. First, to more closely mimic the brain microenvironment *in vitro*, we developed an endothelial cell-neuron co-culture capillary tube-like structure formation assay. In this assay, BECs or mouse dermal microvascular endothelial cells (DECs) were cultured on a bed of rat granule neurons (Figure 5A, B). Under control conditions, BEC’s segregated from neurons and did not form capillary tube-like structures. However, BECs did form capillary tube-like structures within 6 hours of direct VEGF treatment, as expected, or DV treatment. VEGF and DV effects could be inhibited by VEGF neutralizing antibody. Similarly, DECs formed capillary tube-like structures when treated with VEGF, but became more segregated from neurons than under control conditions, where cells did not form tube-like structures, but were more distributed/dispersed among the neurons. In these cells DV treatment also mimicked the effects of direct VEGF treatment. Furthermore, the formation of endothelial tube-like structures was dependent upon the presence of granule neurons. Indeed, in the absence of granule neurons, but with granule neuron-conditioned media, neither VEGF nor DV treated BECs could form tube-like structures [29] (data not shown).

In addition to changes in tube formation, DV promoted BEC proliferation (Figure 5C), which could be blocked with SU1498, an inhibitor of VEGF receptor 2, suggesting that DV’s proliferative effect is also driven through a VEGF-dependent mechanism. Finally, treatment with U0126 also inhibited DV-induced increased proliferation (Figure 5D) denoting the role played by the ERK signaling pathway in DV’s effects on BECs.

## Discussion

**Figure 6:**
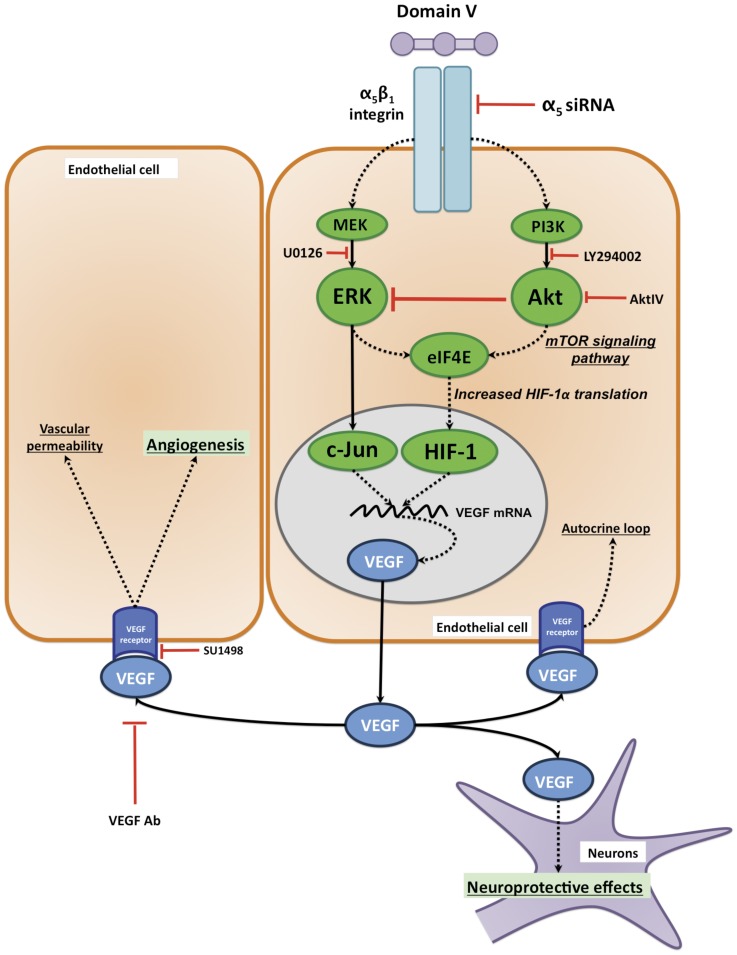
Schematic representation of DV signal transduction, VEGF gene expression, secretion and its subsequent activity at the neurovascular unit. Proposed model by which DV interaction with the BEC α_5_β_1_ integrin results in increased VEGF production and secretion. The use of various antagonists (red lines) is also shown.

Identifying enhancers of endogenous post-stroke brain repair may result in novel stroke therapies. Among the potential brain repair enhancers, VEGF is particularly interesting because of its functional roles in post-stroke neuroprotection, neurogenesis and angiogenesis [30]–[32]. However, direct administration of VEGF acutely after stroke has failed as a therapy, or even worsened stroke damage, largely due to its hyperpermeability effect on the blood-brain barrier [33]–[35]. Indeed, VEGF stroke therapy can be considered to be a “double-edged sword”, inasmuch as acute treatment results in blood-brain barrier breakdown, whereas delayed treatment promotes neuroprotection and repair. Therefore, finding the appropriate therapeutic window in a time- and concentration-dependent manner is essential for considering therapies associated with VEGF. In this study, we investigated the cellular mechanisms by which DV, a bioactive ECM fragment released after stroke [14] leads to VEGF secretion and eventually to its neuroprotective and pro-angiogenic effects [14], as schematized in Figure 6.

### DV Binding to α_5_β_1_ Integrin

Interestingly, DV has demonstrated two opposing effects on angiogenesis explained mostly by an integrin-dependent mechanism: a peripheral anti-angiogenic effect (leading to DV’s other name, endorepellin) driven by α_2_β_1_ [11], and a brain pro-angiogenic effect driven by α_5_β_1_ [14]. In this study, we examined a novel signaling pathway in which DV increases VEGF expression and secretion from BECs. We also further investigated the interaction between DV and the α_5_β_1_ integrin as well as its importance to DV’s pro-angiogenic effect. We hypothesized that DV’s interaction with α_5_β_1_ may occur, at least in part, through DV’s DGR sequence located at the amino acid position 3904 within the EGF2 repeat. The DGR sequence is known to isomerize to an iso-DGR sequence and become a competitive antagonist for RGD ligands [36]. Other studies claim that the iso-DGR sequence is not required for fibronectin binding to α_5_β_1_ [37]. Here, we demonstrated that mutation of DV’s DGR sequence to AGR resulted in a significant decrease in α_5_β_1_ binding and in BEC proliferation, suggesting that the DGR sequence contributes to DV-α_5_β_1_ interaction, but not that DGR is solely responsible for this binding.

Next, as previous reports demonstrated that most of DV's α_2_β_1_ integrin-mediated anti-angiogenic activity outside of the brain was driven via its C-terminal LG3 subdomain, despite LG3 having lower affinity than DV for α_2_β_1_ integrin [11], [16], [38], we investigated LG3’s ability to bind to α_5_β_1_ integrin and influence BEC proliferation. Similar to LG3’s lower affinity for α_2_β_1_, we noted a low, micromolar range K*_d_* for LG3 binding to α_5_β_1_ integrin. However, unlike previous studies demonstrating that lower nanomolar concentrations of LG3 were as or more effective than DV in inhibiting angiogenesis in nonbrain endothelial cells, LG3 was less effective in enhancing angiogenesis (i.e. BEC proliferation) than DV. This seeming contradiction in LG3’s relative potency for anti- versus pro- angiogenic effects, despite similarly low affinities for the implicated DV β_1_ integrin receptor, underscores potential differences in LG3 anti- and pro-angiogenic signaling. Importantly, in our previous work, we noted that LG3 was anti-angiogenic in the human brain endothelial cell line hCMEC/D3 [15]. At that time we speculated that this might be due to differences in LG3’s affinity for α_5_β_1_ integrin. However, we have since determined (Figure S1) that hCMEC/D3 cells surprisingly (and atypically for the majority of microvascular brain endothelial cells) express DV’s anti-angiogenic α_2_β_1_ receptor. As we have previously demonstrated that DV is anti-angiogenic in BECs that normally do not express the α_2_β_1_ via α_2_ integrin plasmid transfection [14], the presence of the α_2_β_1_ integrin in the hCMEC/D3 cell line is likely what accounts for LG3’s previously noted anti-angiogenic effect in these cells.

Collectively, this study suggests that DV’s DGR binding motif plays an important role in DV binding to α_5_β_1_ and hints at a more complex role for DV’s LG3 subcomponent in DV’s pro-angiogenic effects.

### DV Signal Transduction in BECs

In this study, we demonstrate that DV’s activation of ERK-dependent signaling pathways is the primary player in inducing VEGF expression in BECs. Erythropoietin, another important neuroprotective growth factor [39], has also been demonstrated to increase secretion of VEGF by activating Akt- and ERK-dependent pathways. [30], Therefore, the ERK pathway may represent a convergence point for induction of VEGF upregulation. Interestingly, inhibitory crosstalk from Akt to ERK pathways has been previously reported [25]. Our work demonstrates that inhibition of Akt enhances rather than blocks DV’s induction of VEGF, whereas inhibition of ERK completely abolishes DV’s upregulation of VEGF at both the mRNA and protein levels. This suggests that the majority of DV induction of VEGF is through ERK dependent pathways subject to negative regulation control mediated through Akt. These opposing pathways may be partially reconciled by preliminary studies in our laboratory where we observed a significant increase in pAkt, peIF4E and HIF-1α levels under basal conditions in α_5_ knockdown cells, whereas pERK was significantly decreased. These results suggest that α_5_β_1_ integrin conveys a basal ERK-dependent activity that directly or indirectly inhibits an Akt-dependent pathway.

In addition to the activation of these pathways, we demonstrated the ability of DV to transiently stabilize HIF-1α in a normoxic environment. Stabilization of HIF-1α through an O_2_−independent mechanism has been previously described as being mostly driven by activation of the mTOR signaling pathway following Akt phosphorylation [40], [41], which is caused by a globalized increased protein translation in general and of HIF-1α in particular. In additional preliminary studies, we noted an intriguing stabilization of HIF-1α in α_5_β_1_-knockdown cells. Until now, no studies have directly investigated the reason for such stabilization, although a recent study carried out by Ryu and colleagues [42] demonstrated the ability of HIF-1α to up-regulate fibronectin and its cognate receptor, α_5_β_1_ integrin, suggesting a certain relationship between α_5_β_1_ and HIF-1α that will be addressed in more detail in future studies.

This study further highlights the essential role of the α_5_β_1_ integrin for DV signal transduction, and DV’s induction of VEGF in BECs. In addition to an increase of VEGF through an HIF-1α dependent mechanism, we observed c-Jun phosphorylation following DV exposure. This is in agreement with previous literature that demonstrated a direct correlation between increased c-Jun phosphorylation and VEGF expression [43]. Interestingly, other studies have demonstrated a positive cooperative role between HIF-1α and c-Jun [44], [45] suggesting the possibility of a synergistic effect between these two pathways. Specifically, these studies indicate that expression of VEGF through an ERK-dependent pathway, as observed in our study, is strongly amplified by concurrent activation of both HIF-1α dependent and independent (c-Jun) pathways. These findings reinforce the relevance of the signaling pathway detailed here as the main route by which the DV signal is transduced to VEGF production.

### DV and VEGF-mediated Effects on Brain Endothelial Cells

In this study, we demonstrated the relevance of DV-induced VEGF secretion through its biological activity on BECs. We primarily assessed the biological relevance of DV-induced VEGF secretion by investigating changes in endothelial cell proliferation and tube-like structure formation, two important cellular behaviors essential for angiogenesis. We demonstrated in a novel endothelial cell-neuron capillary morphogenesis assay that, consistent with our previous results [14], DV enhances BEC capillary morphogenesis in a VEGF dependent fashion, while inhibiting non-brain capillary morphogenesis as expected. While the possibility that DV influences BEC cell capillary morphogenesis via effects on the neurons present in the assay cannot be ruled out in this co-culture system, it is important to note that preliminary ELISA studies with DV treated neurons did not show increased levels of secreted pro-angiogenic factors including VEGF, BDNF, and NGF (data not shown) suggesting that DV-induced neuronal release of these factors is not a likely cause of DV-enhanced BEC capillary morphogenesis. Likewise, as neither VEGF or DV could induce capillary tube morphogenesis when the granule cells were removed in such a way that their secreted matrix and growth factors remained, the neurons themselves may provide a physical scaffold or platform for intercellular crosstalk for capillary formation under the appropriate circumstances (i.e. in the presence of pro-angiogenic factors such as VEGF or DV). Stated differently, DV, by increasing BEC production and release of VEGF, may foster direct and crosstalk interactions between BECs and neurons that leads to increased angiogenesis. However, it is also important to note that adding endothelial cells to neurons most likely results in changes to the extracellular matrix secreted by the neurons and/or the endothelial cells, which could then in turn contribute to DV’s effects on endothelial cell capillary morphogenesis in the co-culture environment.

In conclusion, we propose the following context for DV action as a novel stroke treatment - Perlecan undergoes proteolysis following cerebral ischemia due to the cellular release of various proteases [4]. Simultaneously, there is an integrin “switch” in brain microvascular endothelial cells from a quiescent state to a pro-angiogenic state that involves an increase in their expression of the pro-angiogenic α_5_β_1_ integrin (and a decrease in the expression of α6β1 integrin) following hypoxia [5]. Our previous results [14] demonstrate that DV both contributes to the increase in brain microvascular endothelial cell α_5_β_1_ integrin expression and directly interacts with the α_5_β_1_ integrin, leading to increased production and release of VEGF. VEGF then plays an important role in DV’s neuroprotective and pro-angiogenic effects following experimental brain stroke in rodents. In the current study, we expanded on previous findings by detailing how DV interacts with the α_5_β_1_integrin, which occurs at least in part, via DV’s DGR amino acid sequence. Finally, we have identified the signaling cascade(s), involved with DV’s induction of VEGF release and linked them with functional significance in brain endothelial cells shedding further light on DV's potential VEGF-mediated ischemic stroke therapeutic mechanism of action.

## Materials and Methods

### Ethics Statement

N/A.

### Cell Culture

Brain microvascular endothelial cells (BECs) from C57Bl6 mice were kindly provided by Dr. Jane Welsh (Texas A&M University) [46] [14]. Cells were grown on gelatin-coated dishes in the presence of Iscove’s Modified Dulbecco’s Medium (IMDM, Invitrogen, Carlsbad, CA) supplemented with 10% Fetal Bovine Serum (FBS, heat inactivated used in all protocols, Sigma-Aldrich, St. Louis, MO) and 1% antibiotic/antimycotic solution (Cellgro, Mediatech Inc, Manassas, VA).

### Recombinant DV Synthesis and Purification

Human recombinant DV was cloned and purified following established protocols [14]. The 26-kDa LG3 C-terminal domain of DV was cloned (Table S1), ligated using the pCep-Pu vector and expressed in HEK-293EBNA cells. LG3 was purified and assessed for purity using similar methods from our previous publication [14]. Mutation of DV at its DGR site (D3904A DV) was done using the Quikchange II XL Site-Directed Mutagenesis Kit (Agilent Technologies, Santa Clara, CA) and confirmed by sequencing. The mutated DV protein was then produced, purified and assessed for purity using Coomassie Brilliant Blue stained SDS PAGE as previously reported [14].

### DV and α_5_β_1_ Integrin Interaction Assays

Binding of DV to α_5_β_1_ integrin was assessed by interaction assay system biosensor (IAsys, Affinity Sensors, UK) following previously described protocols with immobilized α_5_β_1_ integrin (kindly provided by Martin Humphries, U. Manchester)[14], [47]. Free LG3 was added at concentrations ranging from 3.1×10^−8^ ****M to 3.1×10^−7^ M and D3904A DV at concentrations ranging from 8.0×10^−8^ M to 4.0×10^−7^ M. Data from the biosensor were analyzed by the global fitting method described by Myszka and Morton [48]. For each assay, the association rate constants (k*_on_*) and the dissociation rate constants (k*_off_*) were obtained, and the equilibrium dissociation constants (K*_d_*) values were calculated from a ratio of k*_off_*/k*_on_*. In addition, control binding of bovine serum albumin (BSA) at the molar concentration of 8.0×10^−7^ (double of the highest concentration for DV) was also performed.

### Immunoblots

Medium containing 10% FBS was removed from confluent BECs, which were then quickly rinsed one time with pre-warmed (37°C) PBS. The PBS was then quickly removed and replaced with fresh pre-warmed (37°C) medium containing 1% FBS. The cells were serum-starved for 24 h in this 1% FBS containing medium. After 24 hours, this medium was removed and replaced with fresh, pre-warmed (37°C) 1% FBS containing medium. Following two hours of incubation, DV (20 µg/mL) was added directly to the plates (avoiding as much agitation as possible to not induce any artificial signaling) for varying time points at 37°C and 5% CO_2_. For inhibition experiments the same precautions were followed prior to pretreatment of cells for 1 h in the presence of inhibitors at the manufacturer’s recommended doses of 10 µM LY294002 (Cell Signaling Technology, Danvers, MA), 10 µM U0126 (Cell Signaling Technology) or 10 µM AktIV inhibitor (Calbiochem, EMD Chemicals, San Diego, CA), followed by DV treatment. The final concentration of DMSO never exceeded 0.1%.

BECs were washed with ice-cold PBS, homogenized in RIPA lysis buffer (Cell Signaling Technology) complemented with protease inhibitor cocktail (Calbiochem) and centrifuged 10 minutes at 14,000 rpm. Lysate protein concentration was assessed by BCA (Thermo Scientific, Rockford, IL). Samples (20 µg/lane) were loaded onto a 10% SDS-PAGE gel and subsequently transferred to PVDF membranes. Membranes were blocked in 5% non-fat dry milk followed by an overnight incubation at 4°C in the presence of antibodies directed against phospho-Akt (Cell Signaling), pan-Akt (Cell Signaling Technology), phospho-ERK1/2 (R&D Systems, Minneapolis, MN), pan-ERK1/2 (R&D Systems), phospho-eIF4E (Cell Signaling Technology), eIF4E (Cell Signaling Technology), HIF-1α (Novus Biological, Littleton, CA) or glyceraldehyde-3-phosphate dehydrogenase (GAPDH, Sigma-Aldrich). Membranes were washed with TBS-0.1% Tween-20 and incubated in presence of horseradish peroxidase (HRP) conjugated secondary antibody (Genetex, Irvine, CA). Band detection was performed by enhanced chemiluminescent substrate (Picowest Signal, Thermo-Fisher Scientific) and captured by X-ray films. Blot optical density quantification was performed using ImageJ software (ImageJ, NIH, Bethesda, MD). For ERK and pERK, doublet band optical densities for each were added together for quantification.

### qPCR Gene Expression

BECs were washed with ice-cold PBS and total RNA was extracted following 1.5 h DV treatment using RNeasy MiniKit (Qiagen, Valencia, CA). Total RNA was quantified by UV spectrophotomer (Beckman-Coulter, Brea, CA). cDNA were obtained from 1 µg total RNA using AMV reverse transcriptase (Invitrogen). qPCR was performed using TaqMan® Fast Universal PCR Kit (Applied Biosystems, Carlsbad, CA) and appropriate probes (Table S1). ΔCT and ΔΔCT values were calculated using Applied Biosystems 7500 Fast Reverse transcriptase PCR software (Applied Biosystems). GAPDH served as internal control for normalization.

### VEGF ELISA Assay

BECs were serum-starved in 1% FBS containing medium as described above for 24 hours, and then incubated with fresh 1% serum-containing media containing various DV concentrations. Conditioned-medium was collected and spun down at 10,000 g for 5 minutes. VEGF in the conditioned medium was detected using VEGF ELISA kits (Ray Biotech Inc., Norcross, GA) following the manufacturer’s instructions.

### Proliferation Assay

BECs were seeded in 96-well plates at a concentration of 4,000 cells/well in IMDM medium containing 10% FBS. Following overnight incubation, BECs were washed with warm PBS and incubated in serum-free medium (1% FBS) in the presence or absence of 20 µg/mL DV +/− various inhibitors for an additional 24 hours. Following this incubation, 3-(4, 5-dimethylthiazol-2-yl)-5-(3-carboxymethoxyphenyl)-2-(4-sulfophenyl)-2H-tetrazolium solution (MTS, CellTiter 96 Aqueous, Promega, Madison, WI) was added to each well for an additional two hours at 37°C and 5% CO_2_. Changes in MTS coloration were assessed at 490 nm using an absorbance plate reader (Sunrise Phoenix, Tecan, Maennedorf, Switzerland).

### Neuron-endothelial Cell co-culture Tubulogenesis Assay

Cerebellar granule neurons were isolated from the cerebellum of postnatal day 8 rats as previously published [49] generating a >95% pure population. The neurons were then added to an 8 chamber well slide (5×10^4^ cells/well) pre-coated with laminin (Invitrogen) and allowed to grow overnight in Dulbecco’s Modified Eagle Medium (DMEM) containing 10% FBS, 10% horse serum, glucose (6 mM), insulin (10 mg/mL), glutamine (200 mM). The following day, BECs or primary mouse dermal microvascular endothelial cells (DECs, Celprogen, San Pedro, CA) were added to the neuron containing wells (3×10^4^ cells/well) and allowed to grow overnight in DMEM containing serum. Next, cells were vigorously washed with warm PBS to remove serum and further incubated in serum-free DMEM containing 20 µg/mL DV in the presence or absence of VEGF neutralizing antibodies for up to 6 h. VEGF-treated cells were used as positive controls. In some experiments, prior to the addition of the endothelial cells, the neurons were removed following previously published protocols [29] such that their secreted ECM and attached growth factors remained. In other experiments, endothelial cells were added to laminin-coated wells overnight, followed by treatment with VEGF, DV or DV+VEGF neutralizing antibody in cerebellar granule cell conditioned media.

Cells were fixed with 4% paraformaldehyde solution (w/v), permeabilized with 0.1% Triton-X100 and incubated in primary antibodies raised against von Willebrand factor (Dako Cytomation, Denmark) or Tuj-1 (Neuromics, Edina, MN). Cells were washed and further incubated in fluorochrome-conjugated secondary antibodies (Jackson ImmunoResearch, West Grove, PA). Cells were visualized on a confocal microscope (Zeiss, New York, NY). Tube formation was quantified as pixels per high-power field, using Photoshop CS (Adobe, San Jose, CA). Tube formation were measured from 10 random fields per treatment condition, with each treatment condition performed in triplicate per experiment, and three independent experiments performed.

### Statistical Analysis

Data are presented as Mean±SD from a minimum of three independent experiments. Statistical tests were performed using one-way and two-way ANOVA analysis function in Graphpad Prism 4.0 (Graphpad Software, La Jolla, CA). *P*<0.05 was considered to be statistically significant.

## Supporting Information

Figure S1 **Differential expression of α2 integrin in hCMEC/D3 and C57BL6 BEC cells.** Anti-α_2_ integrin (160 kDa) western blot on hCMEC/D3 cells and BECs from C57Bl6 mice, with GAPDH protein (38 kDa) loading control, demonstrating the presence and absence, respectively, of this integrin in these two cell types.
(TIF)

Table S1 **Sequences used for the generation of recombinant proteins and quantitative PCR analysis.** Forward and reverse sequence primers used in this study to generate D3904A mutated domain V (upper lines); PCR primers accession numbers used for the quantification VEGF-A and GAPDH gene expression (lower lines).
(TIF)
