# Supplementary figures and images for: Perlecan Domain V Induces VEGf Secretion in Brain Endothelial Cells through Integrin α5β1 and ERK-Dependent Signaling Pathways
Source: PLoS One. 2012 Sep 17;7(9):e45257. doi: 10.1371/journal.pone.0045257 (PMC3444475; doi:10.1371/journal.pone.0045257)

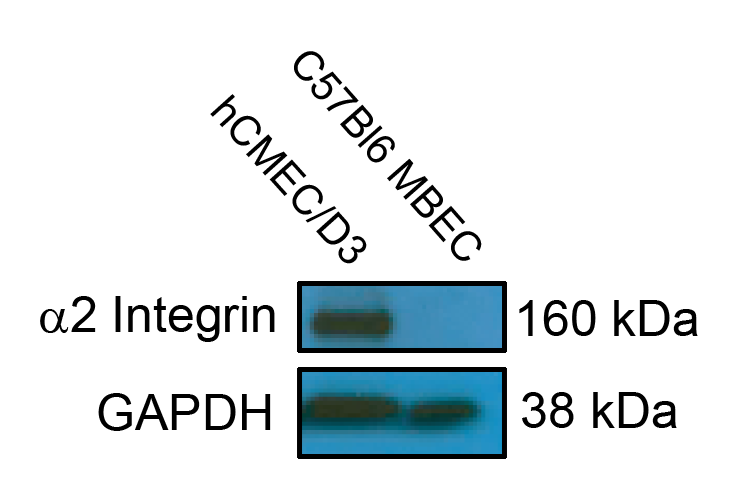

Supplement: Figure S1 — Differential expression of α2 integrin in hCMEC/D3 and C57BL6 BEC cells. Anti-α2 integrin (160 kDa) western blot on hCMEC/D3 cells and BECs from C57Bl6 mice, with GAPDH protein (38 kDa) loading control, demonstrating the presence and absence, respectively, of this integrin in these two cell types. (TIF) [file pone.0045257.s001.tif]

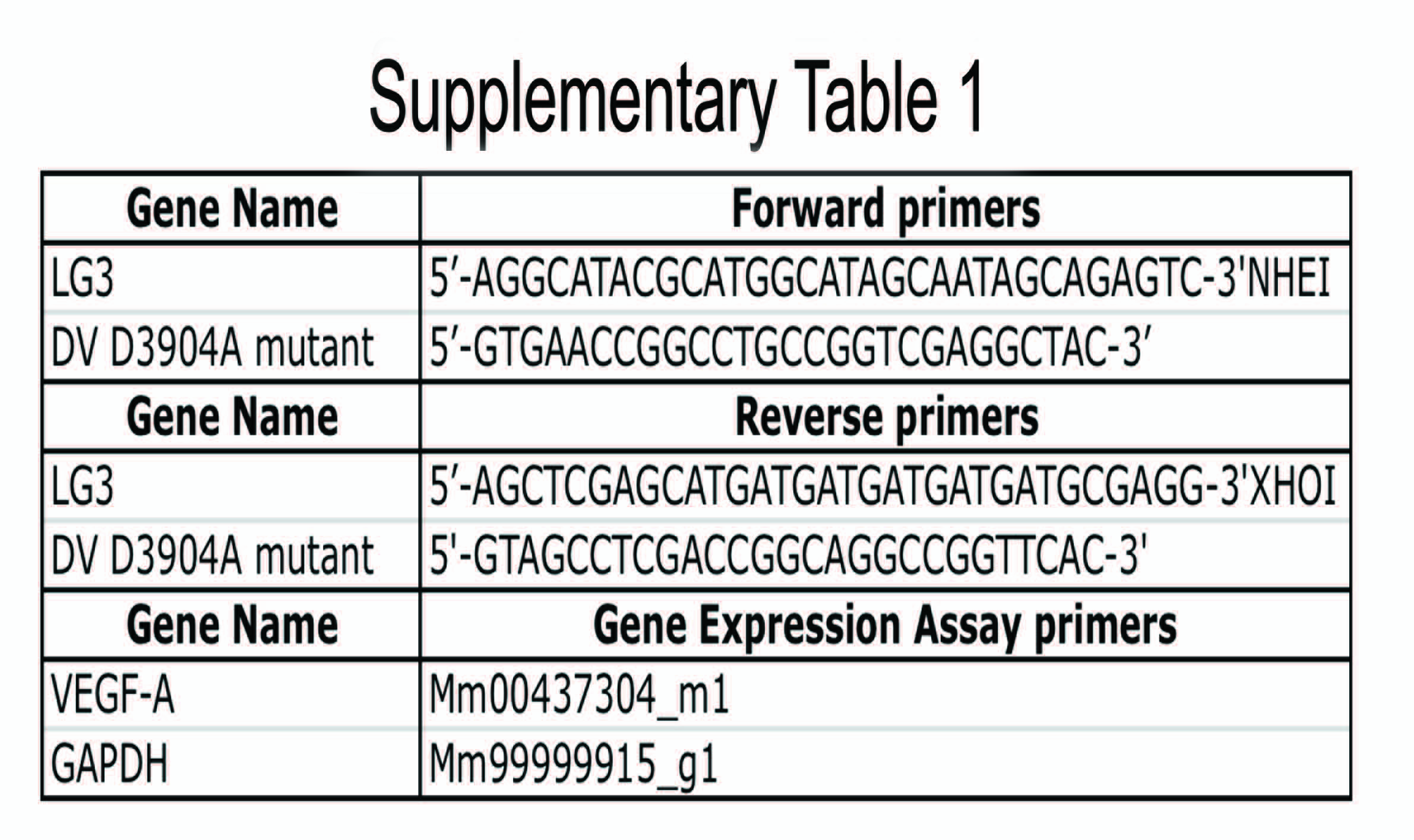

Supplement: Table S1 — Sequences used for the generation of recombinant proteins and quantitative PCR analysis. Forward and reverse sequence primers used in this study to generate D3904A mutated domain V (upper lines); PCR primers accession numbers used for the quantification VEGF-A and GAPDH gene expression (lower lines). (TIF) [file pone.0045257.s002.tif]
